# Polyphenols in Parkinson’s Disease: A Systematic Review of In Vivo Studies

**DOI:** 10.3390/nu10050642

**Published:** 2018-05-19

**Authors:** Małgorzata Kujawska, Jadwiga Jodynis-Liebert

**Affiliations:** Department of Toxicology, Poznan University of Medical Sciences, 30 Dojazd Str., 60-631 Poznań, Poland; liebert@ump.edu.pl

**Keywords:** behavioral tests, rotenone, urolithins

## Abstract

Parkinson’s disease (PD) is the second most common neurodegenerative disorder. However, therapeutic options treating only its symptoms are very disappointing. Therefore there is an ongoing search for compounds capable of tackling the multi-dimensional features of PD. Recently natural polyphenols have gained great interest as potential therapeutic agents. Herein, we have attempted to summarize results obtained in different animal models demonstrating their neuroprotective effects. The in vivo findings presented below are supported by human subject data and reports regarding the ability of polyphenols to cross the blood-brain barrier. The beneficial effects of polyphenols are demonstrated by the results of behavioral examinations, mainly related to motor and cognitive capabilities, histopathological and immunohistochemical examination concerning the protection of dopaminergic neurons, analyses of dopamine and the concentration of its metabolites, as well as mechanistic studies regarding the modulation of oxidative stress, neuroinflammation, cellular iron management, proteinopathy, and additionally the regulation of signaling pathways. Importantly, data about brain distribution of the metabolic derivatives of the reviewed polyphenols are crucial for the justification of their nutritional intake in neuroprotective intervention, as well as for the identification of potential targets for a novel therapeutic approach to Parkinson’s disease.

## 1. Introduction

Parkinson’s disease (PD) is, after Alzheimer’s disease, the second most common human neurodegenerative disorder, which is characterized by motor and cognitive dysfunction associated with the degeneration or loss of dopaminergic neurons in the substantia nigra pars compacta (SNpc). The hallmark lesions called Lewy bodies are produced by the progressive accumulation of protein inclusions containing α-synuclein and ubiquitin in the cytoplasm of selected neurons, which leads to their death by necrosis and/or apoptosis. Despite intensive research, the etiology of PD remains poorly understood. Nevertheless, several underlying pathophysiological mechanisms such as oxidative stress, neuroinflammation, iron dysregulation, mitochondrial dysfunction, excitotoxicity, loss of neurotrophic factors, glial activation, and endoplasmic reticulum stress as well as protein misfolding and dysfunction in their degradation have been credited as significant pathways for the development of therapeutic approaches [1,2]. These complexities of Parkinson’s disease are accompanied by a lack of sufficient therapies, which are limited to long-term symptomatic treatment. In this respect, the concept of neuroprotection referring to the prevention of DA cell death and, hence, to retarding or halting disease progression appears to be a promising strategy. There is an ongoing search for substances targeting the underlying neurodegenerative process. Various substances exhibiting anti-inflammatory, antioxidant, and metal chelating activity in the central nervous system (CNS) have been tested for use to facilitate the management of PD. In this context, natural polyphenols have raised much attention in the recent decade. It has been established that they may exert neuroprotective effects by targeting multiple mechanisms, beyond those above mentioned. Importantly, polyphenols have been reported to inhibit the formation of α-synuclein misfolded aggregates and to reduce mitochondrial dysfunction-induced oxidative stress and inflammatory responses. Moreover, they are capable of affecting cell survival and cell cycle genes as well as signaling pathways. Polyphenolic compounds can activate extracellular signal-regulated kinase (ERK), phosphoinositide 3-kinase (PI3K)/Akt, mitogen-activated protein kinase (MAPK), serine/threonine protein kinase (AKT), protein kinase C (PKC) or inhibit NF-κB pathways. Finally, the antioxidant activity of polyphenols contributes to the activation of the nuclear factor-erythroid 2-related factor 2 (Nrf2)/antioxidant responsive element (ARE) [3,4,5]. These mechanisms have been extensively investigated in in vitro models [6,7,8,9,10,11,12]. However, the findings of the above cited studies cannot be directly translated into in vivo conditions since the action of polyphenols will depend on their biotransformation and the ability to penetrate the blood-brain barrier.

The number of studies concerning neuroprotective properties of polyphenols in animal models is markedly lower than those performed in vitro. Epidemiological and clinical data regarding the relation between polyphenol consumption and the incidence of neurodegenerative diseases, such as PD, are also limited.

The aim of our study was to review the investigation of neuroprotective properties of polyphenols in animal models of Parkinson’s disease, which were supported by human subject data as well as the current knowledge regarding the ability of polyphenols and/or their metabolites to cross the blood-brain barrier. This review does not cover other neurodegenerative disorders.

## 2. Studies in Animal Models

Around 95% of diagnosed Parkinson’s disease cases are sporadic, caused by a combination of environmental exposures and genetic susceptibility as well as aging. In parallel with advances in our understanding of the disease pathogenesis, various animal models of PD have been established. The majority of them are based on environmental contribution to the pathological hallmarks of Parkinson’s disease, including loss of dopaminergic neurons in the SNpc, reduced dopamine (DA) content in the striatum, neuroinflammation and aggregation pathology reminiscent of Lewy bodies as well as motor symptoms. These parkinsonian-like features are developed in animals by the use of neurotoxins targeting the mitochondrial oxidative phosphorylation such as 6-hydroxydopamine (6-OHDA), *N*-methyl-4-phenyl-1,2,3,6-tetrahydropyridine (MPTP), rotenone (ROT) and homocysteine (Hcy). In addition, lipopolysaccharide (LPS), a potent microglial activator, is used to induce inflammatory dopaminergic neurodegeneration. These models facilitate an integrated view of a neuroprotective mechanism in regard to functional improvements with underlying changes occurring in molecular pathways, categories of cells, and brain networks. However, to address a specific molecular target of neuronal degeneration of PD, various genetic models are employed. Since the pathology of PD is multifaceted and several pathomechanisms are involved in the dopaminergic neuronal death, no single model is currently able to recapitulate Parkinson’s disease as seen in humans [1,13].

### 2.1. Pure Compounds

There is an ongoing search for novel therapeutic candidates showing promise for tackling the multi-dimensional features of Parkinson’s disease. Recently, a large number of individual polyphenols have gained considerable interest for their potential in developing alternative drugs or their beneficial effect from nutritional intake [1]. The following sections review the neuroprotective effects of pure polyphenolic compounds. To systematize the great number of reports we have presented them in the order of various PD models. All studies discussed below are summarized in Table 1.

#### 2.1.1. 6−hydroxydopamine Model

The unilateral injection of 6-hydroxydopamine (6-OHDA) into the medial forebrain bundle of rodents has been commonly applied as an experimental model of PD. 6-OHDA generates oxidative stress and induces a lesion of nigrostriatal dopaminergic neurons that results in their progressive loss and consistent behavioral phenotype [81].

Flavonoids represent the most abundant group of polyphenols with well-established anti-parkinsonian effect [82]. Administration of citrus flavanone naringenin to rodents for four days, before lesioning with 6-OHDA resulted in a significant decrease in the loss of tyrosine hydroxylase (TH)—positive cells as well as in the loss of DA and its metabolites 3,4-dihydroxyphenylacetic acid (DOPAC) and homovanillic acid (HVA) in the brain [14,15]. As tyrosine hydroxylase catalyzes the formation of l-3,4-dihydroxyphenylalanine (l-DOPA), which is the rate-limiting step in the biosynthesis of dopamine, thus TH expression is one of the most frequently used markers for identifying DA neurons [83]. Lou et al. [15] studied cellular mechanisms underlying the neuroprotective effects of naringenin, and they have demonstrated that it readily activated Keap1/Nrf2/ARE axis, thereby inhibiting oxidative stress in 6-OHDA-lesioned mice. The pretreatment with glycoside of naringenin − naringin, also has been shown to provide neuroprotection in the experimental model of PD [16]. It protected DA neurons in the mouse brain by reducing neuroinflammation and microglial activation as well as by increasing mammalian target of rapamycin complex 1 (mTORC1) activity [16]. Another flavanone glycoside found in citrus fruits − hesperidin and its aglycone hesperetin were reported to exert neuroprotective effect [17,18]. In the striatum of aged mice, hesperidin attenuated the 6-OHDA-induced reduction in glutathione peroxidase (GPx) and catalase (CAT) activity and total reactive antioxidant potential, leading to increased levels of striatal dopamine and its metabolites and preventing memory impairment and depressive-like behavior [17]. The beneficial effect of hesperetin against 6-OHDA-induced behavioral abnormalities has been demonstrated to be associated with the attenuation of apoptosis, astrogliosis and oxidative stress [18]. However, the neuroprotective effect was not observed after treatment with flavonol quercetin [14]. The lack of quercetin action is in accordance with the results of Kääriäinen et al. [19], who reported that i.p. administration of quercetin for 14 days to rats lesioned with 6-OHDA did not afford protection in TH-positive nigral cell number, striatal fiber density or levels of dopamine. On the other hand, Haleagrahara et al. [20] in the same PD rat model observed increased striatal dopamine and neuronal survivability, which were accompanied by a decreased level of oxidative damage to proteins and lipids and increased content of reduced glutathione (GSH), in rats treated with quercetin, suggesting that it could provide neuroprotection through antioxidant effects. The reported different responses to quercetin could be related to the magnitude of the lesion, which was probably different since Zbarsky et al. [14] and Kääriäinen et al. [19] injected 6-OHDA into the medial forebrain bundle, whereas Haleagrahara et al. [20] did it by an intracisternal puncture. The glycoside of quercetin − rutin, has also been shown to protect against 6-OHDA-induced neurotoxicity in rats via its antioxidant as well as anti-inflammatory activity [21]. Rutin supplementation significantly reduced oxidative damages to membrane lipids and protein levels, and led to an increase in GSH content and the activity of antioxidant enzymes in the striatum of 6-OHDA infused rats. In addition it protected these rats from the rise in circulating tumor necrosis factor alpha (TNF-α) and interleukin 1 beta (IL-1β) production. These effects correlated well with: a decrease in the deficits in locomotor activity and motor coordination, an increase in DA and its metabolite content and an increase in the number of dopaminergic D2 receptors, in lesioned rats pre-treated with rutin [21]. Similar neuroprotective effects of troxerutin, a derivative of rutin were reported [22]. Pretreatment of 6-OHDA lesioned rats with troxerutin improved their motor functions, prevented the loss of nigral TH-positive neurons and decreased the striatal level of lipid peroxidation and ROS. Moreover, the flavonol effectively inhibited astrogliosis and apoptosis. The authors suggested that the beneficial effects of troxerutin could partly be mediated through the PI3K/Akt pathway [22].

Myricitrin, derived from the root bark of *Myrica cerifera*, and its aglycon, flavonol myricetin have also been shown to exert neuroprotective effects. In mice receiving an intrastriatal injection of 6-OHDA, myricitrin improved motor function and protected DA neurons. The beneficial effects were associated with the activation of mTORC1, maintenance of TH activity as well as the anti-neuroinflammatory action [84]. Myricetin prevented the decrease of dopamine and degeneration of TH-positive neurons induced by 6-OHDA in rats. Its protective effects were partially attributed to the suppressed iron toxicity, as was shown by the decreased number of iron-staining cells [23].

The same model of the nigrostriatal lesion was applied to examine the neuroprotective effects of isoflavone genistein given to rats intraperitoneally, 1 h before surgery. Histological study revealed that the compound tested protected nigrostriatal neurons against degenerative effects induced by 6-OHDA. It was supported by the attenuation of rotational behavior induced by apomorphine, the test for nigrostriatal dopamine depletion [24]. Similarly, another isoflavonoid puerarin was demonstrated to reduce the 6-OHDA induced decrease in TH-positive cell counts and to prevent apoptosis. Immunohistochemical analysis proved that puerarin decreased the level of Bax stimulated by 6-OHDA injection. Moreover, puerarin was capable of restoring the content of dopamine and its metabolites, and to enhance the expression level of a glial cell-derived neurotrophic factor (GDNF), which is involved in the survival of adult DA neurons in the striatum [25]. The authors suggested that the latter effect, as well as the inhibition of apoptotic signaling pathways, are the essential events in neuroprotection exerted by puerarin.

Oral administration of flavone found in *Scutellaria baicalensis*, baicalein for one week, re-balanced glutamate (GLu) and gamma-aminobutyric acid (GABA) levels in basal ganglia of 6-hydroxydopamine treated rats resulting in the attenuation of muscle tremor. This effect was caused by regulating the expression of gamma-aminobutyric acid transaminase (GABA-T) and glutamine synthetase (GS)—enzymes involved in the turnover of GLu and GABA as well as by inactivating the neuronal excitation induced by GLu [26].

Administration of tangeretin, a citrus flavanone, to rats p.o. for four days before 6-OHDA lesioning resulted in a significant reduction of the loss in both TH- positive cells and striatal dopamine content [27].

Epigallocatechin-3-gallate (EGCG), a major flavanol in tea, has been widely shown to exert neuroprotective effects in various PD models [85]. However, in 6-OHDA-lesioned rats, only a slight improvement in postural and motor behavior has been observed after oral 14-day pre-treatment with this representative of green tea polyphenols [28].

Intake of the anthocyanin pelargonidin has been reported to dose-dependently attenuate behavioral and structural abnormalities, due to 6-OHDA toxicity and to decrease lipid peroxidation [29].

Some stilbenes were also demonstrated to protect against 6-OHDA-induced neurotoxicity. Resveratrol (RES) given orally for ten weeks reduced neural inflammation in a 6-OHDA-induced PD in rats, as was evidenced by suppression in the production of cyclooxygenase-2 (COX-2) and TNF-α in substantia nigra as well as ameliorating 6-OHDA-induced neurobehavioral deficit [30]. These findings are in agreement with data reported by Wang et al. [31]. They demonstrated that resveratrol administered orally for 14 days decreased the abnormal rotational behavior, the loss and apoptosis of nigral cells, and ROS level, while improving the total antioxidant capacity of nigral tissues significantly in rats injected with of 6-OHDA. In another study, pretreatment with RES for 15 days led to upregulation of the antioxidant status and lowering the dopamine loss in 6-OHDA lesioned rats. That was associated with the attenuation of elevated levels of thiobarbituric acid reactive substances (TBARS) and protein carbonyls (PCs) as well as the activity of phospholipase A2 [32]. Co-treatment with another stilbenoid, piceid, was also found to attenuate 6-OHDA-induced motor defects in rats. Furthermore, the lipid peroxidation level was significantly decreased, and the activity of superoxide dismutase (SOD) was increased after co-administration of piceid. The findings support the tight correlation between the antioxidant activity of polyphenols and their protective effects on motor function [33].

An increasingly popular polyphenol tested for its neuroprotective properties is curcumin (CUR). Long-term pretreatment with this compound reversed the 6-OHDA-induced decrease in the levels of DA and its metabolite DOPAC in the striatum. Moreover, curcumin restored the number of TH-positive neurons and decreased the number of iron-positive cells, as compared with the 6-OHDA treated group [34]. Tripanichkul et al. [35] have shown that the same dose of CUR administered for seven days, starting after 6-OHDA injection, attenuated loss of striatal DA axons triggered by the neurotoxin. Inhibition of microglial and astroglial reaction, as evidenced by reduced glial fibrillary acidic protein (GFAP) and ionized calcium binding adapter molecule 1 (Iba1) immunoreactivity, as well as maintenance of the superoxide dismutase 1 (SOD1) level has been suggested to be one of the mechanisms underlying the neuroprotective effects of curcumin [35].

In rats treated with carnosic acid (CA), a phenolic diterpene found in rosemary, for three weeks before 6-OHDA injection, improvement of the locomotor activity and reduction of apomorphine-caused rotations have been observed. Moreover, pretreatment with CA provided protection against lipid peroxidation and GSH depletion in the substantia nigra. Those effects were observed concurrently with the increase in the protein expression of c-glutamate-cysteine ligase, superoxide dismutase, and glutathione reductase in the striatum. Finally, CA has been demonstrated to reverse the 6-OHDA-induced activation of c-Jun NH2-terminal kinase and p38, the down-regulation of the Bcl-2/Bax ratio, the up-regulation of cleaved caspase-3/caspase-3 and cleaved PARP/PARP ratios, and the down-regulation of tyrosine hydroxylase protein in the striatum of rats. These results suggest that the CA protective effect against 6-OHDA-induced neurotoxicity is attributable to its anti-apoptotic and anti-oxidative action [36].

Zare et al. [37] demonstrated the neuroprotective potential of sinapic acid, hydroxycinnamic acid widespread in plants, given to rats p.o. before injection of 6-OHDA as evidenced by the improved turning behavior, reduced loss of dopaminergic neurons in the substantia nigra, lowered iron reactivity, and decreased the level of MDA and nitrite [37]. Ellagic acid, another natural polyphenol antioxidant found in numerous fruits and vegetables, administered by gavage caused an improvement in locomotion and a reduction in the levels of neuroinflammatory biomarkers, TNF-α and IL-1, in the striatum and hippocampus of rats treated with 6-OHDA [38].

Studies on cannabinoids representative, delta 9-tetrahydrocannabinol, revealed that daily administration (i.p.) of the compound for two weeks to rats produced a significant attenuation in the magnitude of the following changes evoked by 6-OHDA injection: a depletion of dopamine content and a reduction in tyrosine hydroxylase activity in striatum, accompanied by a reduction in tyrosine hydroxylase mRNA levels in the substantia nigra [39].

#### 2.1.2. *N*-methyl-4-phenyl-1,2,3,6-tetrahydropyridine Model

Another toxin employed as an experimental model to study therapeutic strategy against PD is *N*-methyl-4-phenyl-1,2,3,6-tetrahydropyridine (MPTP), which induces dopaminergic degeneration via oxidative stress [81]. The active metabolite of MPTP − MPP^+^, is selectively taken up by dopaminergic neurons and actively transported into mitochondria where it interferes with the respiratory chain and inhibits complex I, inducing energy depletion and ROS production. The MPTP-induced neurotoxicity is limited to the nigrostriatal dopaminergic pathway and is associated with motor dysfunctions [13].

This model was applied broadly to examine the neuroprotective properties of EGCG, one of the main active components of green tea [40,41,42,43,44,45,46]. Treatment with EGCG has been demonstrated to prevent MPP^+^-induced motor impairment [43,46] and dopamine neuron loss [40,41,42,45,46], and depletion of DA [40,41,42,43,44] in the substantia nigra. Moreover, EGCG has been shown to increase expression and activity of tyrosine hydroxylase [40,41]. Mandel et al. [41] demonstrated that EGCG prevented an MPTP-induced increase in the number of synuclein-positive neurons in the substantia nigra, upregulation of Bax and the depletion of protein kinase C (PKCα) in the striatum. It has also been suggested that the decrease in the expression of neuronal [42] and inducible nitric oxide synthase [45] in the SNpc and striatum in MPTP-treated mice has contributed significantly to the neuroprotective effect of EGCG. Another study showed that EGCG caused restoration of the MPTP-reduced level of the survival factor Ras [44]. Nigral iron accumulation has been demonstrated in animals treated with MPTP that may be associated with the altered expression of iron-related proteins, such as the increased expression of iron importer divalent metal transporter 1 (DMT1), or the decreased expression of the iron exporter ferroportin [86,87,88]. EGCG post-treatment for seven days has been reported to regulate the iron-export protein ferroportin in the SNpc and to reduce oxidative stress in mice. In addition, EGCG restored MPTP-induced functional and neurochemical deficits (DA and DOPAC) to a value similar to the control group [43]. Besides the influence of EGCG on the brain Zhou et al. [46] have demonstrated that its protective effect against dopaminergic neurons loss is also associated with modulation of peripheral inflammation. The authors have reported that EGCG treatment caused a decrease in the ratio of CD3^+^CD4^+^ to CD3^+^CD8^+^ T cells in peripheral blood and in concentrations of serum TNF-α and IL-6 of MPTP-treated mice [46].

In MPTP-treated mice, co-administration with natural flavone acacetin for three days (p.o.) caused a decrease in time of turning, and locomotor activity as compared to those in MPTP-only-treated mice. In the acacetin co-treated group, the inhibition of MPTP-induced degeneration of DA neurons and depletion of dopamine level in the substantia nigra and striatum of the brain were observed. Moreover, administration of acacetin inhibited microglial activation accompanied by decreased production of inducible nitric oxide synthase (iNOS) and COX-2 [47]. Baicalein administration improved the behavioral performance in the motor balance test in mice injected with MPTP. The effect was associated with the attenuation of the MPTP upregulated upsurge of glutamatergic strength that might result from the suppression of cytokine production, including IL-1β and TNF-α in the substantia nigra and striatum [48]. Similarly, in the study by Lee et al. [49], baicalein was shown to improve motor ability, prevent dopaminergic neuron loss and reduce microglial and astrocyte activations caused by MPTP. Two-week administration of 7,8-dihydroxyflavone to mice (i.p.) attenuated the neurotoxic effects of MPTP as evidenced by the preservation of tyrosine hydroxylase expression and the reduction of activated caspase-3. The compound tested has been recognized as a high-affinity agonist of tyrosine kinase receptor B that provokes downstream signaling similar to the action of a brain-derived neurotrophic factor, which mediates neuronal survival, synaptic plasticity, and neurogenesis [50]. Takano et al. [51] reported a significant reduction in the loss of both TH- positive cells and striatal dopamine content in mice given another citrus flavonone, tangeretin i.p., for four consecutive days post MPTP injection. Administration of nobiletin, a polymethoxylated flavonoid isolated from citrus fruit peel, improved MPTP-induced motor dysfunction and cognitive deficits in C57/BL6N mice. The effect was correlated with enhanced activities of striatal and hippocampal Ca^2+^/calmodulin-dependent protein kinase II (CaMKII) and cAMP-dependent protein kinase (PKA), which are critical for TH activity [53]. Jeong et al. [52] have demonstrated that nobiletin protected DA neurons and attenuated interleukin-1b production by inhibiting microglial activation and preserving GDNF expression in the SNpc. Similarly, it has been found that intraperitoneal injection of a flavanone glycoside naringin, prevented nigrostriatal DA neurons against the MPP^+^-induced degeneration through the induction of glial-derived neurotrophic factor (GDNF). Moreover, the naringin attenuated level of TNF-α in microglia increased after exposure to MPP^+^ [54].

Flavonol kaempferol has been found to improve motor coordination, to raise striatal dopamine and its metabolites levels, to increase SOD and GPx activity, and to reduce the content of MDA compared with mice treated with MPTP alone. Immunohistochemical studies showed that treatment with kaempferol could prevent the loss of TH-positive neurons induced by MPTP [55]. Similarly, another flavonol, quercetin, protected against oxidative stress by increasing SOD and GPx activity and against dopamine depletion as well as improved motor balance and coordination. The compound also counteracted inhibition of acetylcholinesterase (AChE) activity and maintained the resting membrane potential of neurons in MPTP-treated mice [56]. Neuroprotective activity of morin, a flavonol originally isolated from the *Moraceae* family, was investigated in mice administered with the compound i.p. for ten days concomitantly with MPTP given for the last five days. Morin significantly attenuated striatal dopamine depletion and a loss of TH-positive neurons caused by MPTP treatment. Moreover, as evidenced by the measurement of cataleptic time (bar test) and the number of steps (drag test), behavioral deficits induced by neurotoxin were also reduced [57].

A similar protective effect on the motor deficit and dopaminergic neuronal loss caused by MPTP has been reported in mice co-treated with silibinin (SIL), a flavonolignane derived from *Silybum marianum* [58]. The authors concluded that the neuroprotective effects of SIL were mediated by the stabilization of neuronal mitochondrial transmembrane potentials (MMPs) [58]. In another study, treatment with silibinin ameliorated in a dose-dependent manner the MPP^+^-induced neurotoxicity in the SNpc of rats, as was clearly shown by an increase in TH-positive cell number. In addition, treatment with SIL attenuated the MPP^+^-induced levels of inflammatory molecules such as TNF-α, IL-1β, and iNOS [59]. Co-treatment with SIL for seven days significantly protected against the progression of MPP^+^-induced striatal dopaminergic neurotoxicity in rats as evidenced by attenuation of motor deficits. This action was mediated through the increase in DA level in the striatum. SIL treatment improved striatal mitochondrial function and integrity as well as the enhanced activity of SOD. Anti-apoptotic and anti-inflammatory properties of SIL, as demonstrated by a decrease in expression of caspase-3 and NFκB protein, might also contribute to its neuroprotective activity [60].

Genistein pretreatment reduced neurotoxicity in MPTP-challenged mice, which was attributed to increased contents of DA, DOPAC, and HVA in striatum and number of TH-positive neurons in the SNpc. This phytoestrogen restored the decrease in MPTP-induced Bcl-2 gene expression in mice midbrain and therefore positively regulated cell survival [61].

Gastrodin, the phenolic glucoside from *Gastrodia elata*, showed neuroprotective effects in the subchronic MPTP mouse model of PD. It was evidenced by ameliorating bradykinesia and motor impairment as well as by its beneficial effects against dopamine depletion and by a reduction in reactive astrogliosis caused by MPTP in the substantia nigra and striatum of mice. Moreover, gastrodin efficiently prevented neuronal apoptosis through the reduction in the protein expression of proapoptotic Bax and caspase-3 protein and the increase in the antiapoptotic Bcl-2 expression. As a consequence, a MPP^+^-induced activation of the downstream target of caspase-3-poly (ADP-ribose) polymerase (PARP) contributing to cell death, was substantially suppressed [62].

Phenanthrenequinone tanshinone I, a constituent of *Salviae miltiorrhizae*, also improved motor functions, normalized striatal level of DA, and provided dopaminergic neuronal protection in mice receiving four i.p. injections of MPTP-HCl, as evidenced by the increased number of TH-positive neurons. The antiparkinsonian effect of tanshinone I was mediated by the modulation of microglial activation leading to the attenuation of the increase in TNF-α and IL-10 concentrations [63].

Ferulic acid (FA), hydroxycinnamic acid abundant in plants, administered for seven days to MPTP injected mice attenuated the formation of MPP^+^ and altered Bax/Bcl-2 ratio exerting an antiapoptotic effect in the substantia nigra region. The authors of the report suggested that both the antioxidant and antiapoptotic effects of FA contributed to the improvement of motor balance and coordination in mice challenged with MPTP [64].

Madecassoside, a saponin isolated from *Centella asiatica*, administered intragastrically for seven days, was effective in recovering MPTP-induced early signs of parkinsonism, as evidenced by improved locomotor dysfunction as well as the protection of dopaminergic neurons and dopamine loss in the striatum. The observed effects were accompanied by a decrease in MDA content and an increase in the GSH level, Bcl-2/Bax ratio, and protein expression of brain-derived neurotrophic factor (BDNF) [65].

Curcumin was demonstrated to attenuate MPTP-induced toxicity when administered to mice i.p. immediately post MPTP injection [66] or for seven days [67]. MPTP-induced depletion of striatal dopamine was markedly limited [66,67], the density of dopaminergic neurons was increased, and the MAO-B activity was inhibited [66] as a result of curcumin treatment. Recently, it has been reported that pre-treatment with curcumin derivatives significantly protected against MPTP-induced dopaminergic neurodegeneration, as assessed by attenuation of striatal DA and DA turnover as well as by a significant decrease in the loss of TH immunoreactivity [68]. Moreover, the authors have revealed that MPTP-induced inflammatory responses, as manifested by stimulated GFAP protein expression and elevated pro-inflammatory cytokines in the striatum of mice brains, were markedly suppressed by curcuminoids treatment.

Ginsenoside Rg1, a triterpenoid saponin found in ginseng species, injected to mice i.p. for eight days reduced the loss of dopaminergic neurons caused by MPTP, prevented glutathione depletion, and caused an increase in SOD activity in the substantia nigra region. As the JNK signaling cascade contributes to dopaminergic neuron apoptosis, the observed attenuation of the phosphorylation of JNK and c-Jun in the substantia nigra, as well as antioxidant activity of ginsenoside, play a significant role in its neuroprotective effects [69].

Daily oral treatment of mice with piceid, for 14 days starting from the first day of subcutaneous injection of MPTP, significantly prevented motor deficits as well as preserved SOD activity and reduced MDA level in the striatal region. Furthermore, piceid protected TH positive DA neurons and activated survival signaling (e.g., increased p-Akt expression and reduced activated caspase-3) [33].

In another model of PD, MPTP is given with probenecid (p) that blocks the renal clearance of MPTP to prolong its action [89]. Several studies using this model were performed for evaluating the antiparkinsonian effects of theaflavin (TF), a black tea polyphenol. Oral pretreatment of MPTP/p administered mice with TF for 35 days caused normalization of their motor function. The effect was accompanied by attenuation of neurodegeneration and apoptosis, as evidenced by an elevated expression of nigral TH and dopamine transporter (DAT) as well as the reduced level of apoptotic markers such as caspase-3, 8, and 9 [70]. Moreover, TF has been found to increase striatal GSH level and GPx activity in MPTP-treated mice [71]. The neuroprotective effect of theaflavin has also been demonstrated to depend on an inhibition of the release of pro-inflammatory cytokines as well as on a decrease in the expression of proapoptotic Bax and an increase in the expression of antiapoptotic protein Bcl-2 in the striatum, which processes counteract the initiation of apoptotic events [72].

Flavones apigenin and luteolin given orally for 26 days, including five days of pretreatment, have been demonstrated to exert multifunctional antiparkinsonian effects like the protection of tyrosine hydroxylase expression, alleviation of oxidative stress and inflammation, enhancement of neurotrophic activity leading to improved motor coordination and locomotor behavior in an MPTP/p mouse model. Additionally, treatment with the compounds tested caused a decrease in astrocyte activation, reflected by the reduced expression of the GFAP in the SNpc of the brain. The authors suggested that the neuroprotective effect of both flavonoids has been attributed to the enhancement of neurotrophic potential as evidenced by the increased level of the brain-derived neurotrophic factor (BDNF) in the substantia nigra region [73].

Syringic acid (SA) is one of the major naturally occurring benzoic acids exhibiting strong antioxidant activity. Oral pre-treatment of mice with SA (1 h before each MPTP injection) for 35 days was found to improve the MPTP/p-induced impaired motor functions by restoring the dopamine content as well as to enhance the antioxidant defense system in the brain. In addition, SA showed the ability to ameliorate MPTP/p-induced decrease in expressions of TH, DAT, and vesicular monoamine transporter-2 (VMAT2) and to attenuate the production of pro-inflammatory cytokines, such as TNF-α, IL-1β, and COX-2 [74].

#### 2.1.3. Rotenone Model

Rotenone (ROT), another inhibitor of respiratory complex I, is also used to induce dopaminergic neuronal oxidative damage in experimental animal models of PD. Rats exposed to prolonged, low-dose rotenone treatment develop a PD-like phenotype characterized by motor dysfunction, a loss of dopaminergic neurons, the formation of Lewy-like inclusions, and microglial activation [90].

In a rotenone model of PD, the application of quercetin to rats for four days rescued motor dysfunction by enhancing dopamine release in the striatum and prevented the loss of nigral TH-positive neurons. In addition, this flavonol attenuated the ROT-induced loss of mitochondrial complex-I activity and GSH depletion as well as blocking programmed cell death in nigral neurons [75].

Curcumin administered for 50 days significantly alleviated motor dysfunction and increased TH activity in the SNpc of ROT-injured rats. The biochemical assessment indicated that in rats pretreated with curcumin an elevated level of GSH and a decreased content of reactive oxygen species and malondialdehyde were observed. Mechanistic studies demonstrated that curcumin was able to restore levels of heme oxygenase-1 (HO-1) and NAD(P)H: quinone oxidoreductase 1 (NQO1) expression and to activate Akt/Nrf2 signaling pathway, thus ameliorating ROT-induced damage in the brain [76].

In rats given rotenone subcutaneously for five weeks, co-administration (p.o.) of piceid attenuated rotenone-induced motor defects in a dose-dependent manner. The dose of 80 mg/kg/day caused an even better effect than l-levodopa (l-dopa)—the gold standard for treating Parkinson’s disease. Treatment with piceid prevented the rotenone-induced changes in the levels of GSH, thioredoxin, ATP, MDA, and SOD activity in the striatum. Furthermore, treatment with the stilbenoid rescued rotenone-induced dopaminergic neurodegeneration in the substantia nigra region [33].

Ferulic acid co-administered to ROT-treated rats for four weeks rescued DA neurons in substantia nigra area and nerve terminals in the striatum from rotenone damage. FA restored the activity of antioxidant enzymes and GSH content while inhibited lipid peroxidation. Following treatment with FA, the production of inflammatory mediators such as COX-2 and inducible NOS and proinflammatory cytokines was also reduced. In addition, these effects were supported by a reduction in expression of Iba1 and GFAP suggesting attenuation of microglial and astrocytic activation. Hence, it could be concluded that protective effects of FA were mediated through its antioxidant and anti-inflammatory properties [77].

Another study has demonstrated that sesamol, a phenolic compound found in sesame seeds, and naringenin, given orally for ten consecutive days, had protective effects in rotenone-lesioned rats. It was evidenced by improved motor skills and enhanced expression of neuroprotective proteins, including parkin, PARK 7 protein (DJ1), C terminus Hsp70 interacting protein (CHIP), and TH in the striatum and substantia nigra. These results correlated well with the reduction in caspase-9 and 3 and ubiquitin levels. Moreover, in the same rats, improved morphology and survivability of neurons were noticed [78].

#### 2.1.4. Lipopolysaccharide Model

Another toxin-induced PD model consists in the treatment of rodents with lipopolysaccharide (LPS), a potent microglial activator, which is able to induce toxicity to dopaminergic neurons. Pretreatment of rats with EGCG 24 h before LPS intraperitoneal injections for seven days was found to have an anti-inflammatory effect through reducing the level of TNF-α and NO as well as to preserve the DA level in the midbrain [79].

#### 2.1.5. Homocysteine Model

In epidemiological and longitudinal studies, a tight correlation between homocysteine (Hcy) and cognitive impairment due to apoptosis, neuronal death, oxidative stress, overactivation of glutamate receptors, mitochondrial dysfunctions, and activation of caspases for all of the neurodegenerative diseases has been demonstrated [80]. Intranigral infusion of homocysteine in rats is used to inhibit mitochondrial complex-I activity and to reduce striatal dopamine in order to induce neurodegeneration in SNpc and impaired motor activity [91]. Mansouri et al. [80] have investigated the neuroprotective effects of curcumin against Hcy-induced neurotoxicity in substantia nigra in the rats. They have found that administration (i.p.) of curcumin for ten days, beginning five days prior to homocysteine intracerebroventricular injection caused the improvement of locomotor function. The effect was accompanied by a reduction in HCy-induced apoptosis and an increase in Bcl-2 as well as a decrease in Bax proteins expression [80].

### 2.2. Beverages and Extracts

There is evidence that plant extracts often have greater activity than isolated constituents at a comparable dose, which is due to pharmacodynamic and/or pharmacokinetic synergy. In addition, the polyphenol–gut microbiota interactions and effects of food matrix on gut microbiomic profile and integrity have been demonstrated to contribute to the health effects of this class of compounds [92,93]. Studies concerning the comparison of the neuroprotective properties of extracts and pure compounds are very scarce. The single example is the report of Wu and Zhu [94] who revealed that contrary to the neuroprotective effect of *Gingko biloba* extract (EGb1), its two components ginkgolides A and B did not demonstrate any activity in the same experimental protocol.

The cocktail drink EM-X derived from the fermentation of rice, papaya, and seaweeds with effective microorganisms was examined in the 6-OHDA rat model of PD. The material tested was rich in lycopene, ubiquinone, various saponins as well as polyphenols, such as quercetin and kaempferol. Four-day administration of the test drink to rats treated with 6-OHDA, instead of drinking/tap water, attenuated the loss of TH- positive cells and the reduction of dopamine and its metabolite HVA levels in the SNpc [95].

*Ginkgo biloba* extracts have been used for many decades to increase cerebral blood flow and to treat dementia. The main active substances of *G. biloba* contributing to its neuroprotective effects belong to two groups of compounds, the flavone glycosides (quercetin, kaempferol, and isorhamnetin glycosides) and the terpene lactones (ginkgolides A, B, C, J, and bilobalide) [96]. In an early study by Wu and Zhu [94] the neuroprotective effects of a standardized extract of Ginkgo biloba (EGb 761) were investigated in mice lesioned with repeated administration of MPTP. Two-week administration of the extract tested (20, 50, 100 mg/kg/day, i.p.; 7 day pre-treatment and 6 day co-treatment) significantly protected striatal dopamine level [94]. The same extract was administered i.p. to rats for a week before 6-OHDA striatal injection and one week after surgery (50 or 100 mg/kg/day). Behavioral deficits induced by 6-OHDA were attenuated as well as dopamine neuron loss in the SNpc, and depletion of striatal dopamine were significantly reduced in rats treated with EGb 761 [97]. In the experimental model using MPTP, *Gingko biloba* extract administered to rats for 20 days (i.p., 50 or 100 mg/kg/day) was demonstrated to improve behavioral deficits, to reduce the decrease in dopamine level and SOD activity in the SNpc and to suppress the level of lipid peroxidation [98]. More parameters were assayed in the experiment involving 3-week pretreatment of rats with *Gingko biloba* extract (EGb) (50, 100, 150 mg/kg/day; p.o.) before a lesion induced by 6-OHDA. Beside the improvement of behavioral deficits, the extract dose-dependently restored the glutathione level, antioxidant enzymes activities, and lipid peroxidation level in the SNpc. The 6-OHDA-induced decrease in dopamine and its metabolites levels and the increased number of dopaminergic D2 receptors in the striatum were restored significantly following EGb treatment. Consequently, with the changes in these parameters, immunohistochemical analysis revealed a substantial increase in the density of TH-IR fibers in the substantia nigra [99].

Indisputably, green tea polyphenols (GTPs), including (−)-epigallocatechin gallate (EGCG), (−)-epicatechin gallate (ECG), (−)-epigallocatechin (EGC), and (−)-epicatechin (EC) have been one of the most extensively studied polyphenols pertaining to neuroprotective effects and not only in PD models. Seven-day pretreatment with GTPs (150 and 450 mg/kg/day; p.o.) was shown to protect midbrain and striatal dopaminergic neurons from 6-OHDA-induced cell death in rats. The effect correlated well with the decrease in the level of nitric oxide synthase (NOS), the content of reactive oxygen species (ROS) and nitric oxide (NO) as well as with attenuation of lipid peroxidation and enhancement of the free radical scavenging capability within the nigrostriatal region [100]. In MPTP-lesioned monkeys, oral administration of tea polyphenols (TPs), containing mainly EGCG, post to MPTP administration in a dose of 40 mg/kg/day for 80 days, caused the reduction in striatal synuclein oligomers’ level, an increase in the number of nigral TH-positive neurons and in the levels of DA and its metabolites DOPAC and HVA in the striatum, which was found to correlate well with improved motor deficits [101].

In another study, silymarin, a complex of flavonolignans derived from the seeds of *Silybum marianum*, has been demonstrated to exert neuroprotective effects through its antioxidant and anti-inflammatory properties. Silymarin treatment (50 and 100 mg/kg; i.p.) for 5 days after MPTP intoxication preserved dopamine levels as well as significantly diminished the number of apoptotic cells and preserved dopaminergic neurons in the substantia nigra region in mice [102]. Singhal et al. [103] examined neuroprotective properties of silymarin in a long-term experiment using another PD model involving two pesticides, maneb, and paraquat (MB + PQ). Silymarin was administered in a dose of 40 mg/kg (i.p.) to mice for 9 weeks, simultaneously some subsets of animals received maneb and paraquat i.p. twice a week. Silymarin treatment significantly attenuated MB+PQ-mediated reduction in locomotor activity, dopamine content, TH-immunoreactivity, and vesicular monoamine transporter (VMAT2) mRNA expression. Moreover, the substance tested reduced the number of degenerating neurons and restored the level of lipid peroxidation, nitrite content, mRNA expression of CYP2E1 and GST A, catalytic activities of CYP2E and GST as well as the expression of P-p53 and caspase-9 proteins. Hence, the authors concluded that silymarin could afford dopaminergic neuroprotection by the modulation of oxidative stress and apoptotic pathways [103].

The protective effects of *Polygonum multiflorum* extract (containing stilbenes, anthraquinones, and flavonoids) on the degeneration of nigrostriatal DA neurons induced by a combination of paraquat and maneb were investigated in a 6-week experiment on mice. Administration of the extract for 47 days simultaneously with neurotoxins (400 and 800 mg/kg/day; p.o.) significantly attenuated an impairment of behavioral performance and a decrease in the striatal dopamine level as well as in substantia nigra tyrosine hydroxylase-positive neurons [104].

Various extracts were investigated in the rat model of PD: tangerine peel rich in polymethoxylated flavones (35 mg/kg/day), cocoa rich in procyanidins (100 mg/kg/day), red clover rich in isoflavones (200 mg/kg/day), and grape seeds rich in catechins (100 mg/kg/day). Rats were treated p.o. for four days with the extracts, and the nigrostriatal lesion was induced by infusing 6-OHDA. Classical parameters of PD, namely the number of the dopaminergic cells in the SNpc and the levels of dopamine and its metabolites DOPAC and HVA in the striatum were quantified. Pretreatment of rats with the extracts of tangerine peel, cocoa, and red clover significantly attenuated the 6-OHDA-induced dopaminergic loss. No marked protection was seen in animals supplemented with grape seeds containing catechins [105].

Five-day pretreatment with a proanthocyanidin-rich fraction from the bark of *Croton celtidifolius* (10 mg/kg/day; i.p.) prevented mitochondrial complex-1 inhibition and a decrease in the expression of tyrosine hydroxylase in the striatum and olfactory bulb in rats given a single intranasal dose of MPTP. Moreover, the extract attenuated the short-term memory deficits, depressive-like behavior and a reduction in locomotor activity observed after MPTP administration. Catechins and oligomeric proanthocyanidins (formed of catechin and epicatechin units) are the main active substances found in the extract tested [102]. The authors suggested that besides antioxidant and ferric iron chelation properties the compounds of catechol-like structure inhibit the uptake of MPP^+^, the toxic metabolite of MPTP, and protect dopaminergic neurons. Additionally, these compounds inhibited monoaminooxidase B (MAO B), the enzyme catalyzing the oxidative deamination of monoamines including DA, thus decreasing its turnover, which resulted in the reduced formation of hydrogen peroxide and the limited generation of MPP^+^ from MPTP [106].

A polyphenol-rich aqueous walnut extract (JSE; an extract of *Juglandis Semen*), standardized based on caffeic acid content, has been demonstrated to protect dopaminergic neurons and to prevent the reduction of striatal DA and its metabolites, when it was given in a dose of 100 mg/kg/day (p.o.) for 6 days in mice exposed to MPTP. Moreover, the JSE treatment inhibited MPTP-induced PD-like symptoms, including reduced ambulation, bradykinesia, impaired motor coordination, and postural balance. The authors of this study suggested that JSE partly prevented the DA loss due to its MAO inhibitory and antioxidant activities [107].

Combined treatment with two polyphenols protocatechuic acid (PCA) and chrysin from the fruits of *Alpinia oxyphylla* at a dose of 10 mg/kg significantly attenuated both MPTP and 6-OHDA-induced dopaminergic neuron loss and ameliorated motor deficiencies in mice [108].

## 3. Epidemiological Studies

Data on dietary and lifestyle factors associated with PD are very sparse, especially in relation to dietary intake of polyphenols.

A large prospective study carried out over two decades involving almost 130,000 individuals showed that men with a high habitual intake of flavonoid-rich food and beverages were at a lower risk of PD. The association was confirmed for greater anthocyanin and anthocyanins-rich berries and apple intake and lower PD risk [109].

The early report concerning the relation of tea drinking habit to PD was published by Chan et al. [110]. Groups of 215 patients with PD and 313 controls in a Hong Kong Chinese population were enrolled in the study. It was demonstrated that black tea drinking reduced the risk of PD [110].

Further studies confirmed these findings. A case-control study conducted in Washington State in 1992–2000 involved 210 PD cases and 347 controls. Reduced risks of PD were observed for consumption of 2 cups or more of tea a day (relative risk = 0.4, 95% confidence interval: 0.2–0.9) and 2 or more cola drinks a day (relative risk = 0.6, 95% confidence interval:0.3–0.1.4) [111].

In a case-control study in a Chinese population, 310 PD patients and 500 controls were screened. It was found that 3 cups/day of tea for ten years led to a 28% reduction of PD [112].

Another study conducted in 1993–2005 on Chinese population involved 157 incident PD cases. Black tea intake showed an inverse association with PD risk (relative risk = 0.29, 95% confidence interval: 0.13–0.67) and ingredients other than caffeine were responsible for this effect. Contrary to numerous in vitro findings, green tea drinking was unrelated to PD risk [113]. The data on the inverse correlation between long-term consumption of tea and the onset of PD have been reported by Kandinow et al. [114]. Tea intake of at least 3 cups per day has been demonstrated to delay the motor symptoms onset of PD even by 7.7 years [114]. Other authors observed that consumption of one cup of tea per day or more dose-independently exerts neuroprotection in PD [115]. Finally, a meta-analysis study of tea drinkers and non-drinkers showed that the first ones have lesser risk being affected by PD [116]. This protective effect has been suggested to be attributed to the antioxidant, anti-inflammatory, and anti-excitotoxic activity of tea polyphenols [117].

## 4. The Permeability of Polyphenols Across the Blood-Brain Barrier

Despite evidence for the neuroprotective properties of polyphenols, information concerning their ability to enter CNS is limited. Since ingested polyphenols are extensively metabolized, it is critical to understand their metabolism as well as bioavailability and brain distribution of their metabolites to confirm the beneficial effects in PD.

To evaluate the ability of various compounds to gain entry to the brain, the in vitro blood-brain barrier (BBB) model consisting of brain endothelial cell lines and ECV304 monolayers co-cultured with C6 glioma cells was established. This cells culture showed upregulation of some features characteristic of the BBB in vivo including increased tight junction organization and elevated TERR. Using this model, the permeability of hesperetin, naringenin, and their respective glucuronides, as well as anthocyanins C3R and P3G across BBB, was demonstrated [118].

The above mentioned in vitro model does not fully mimic physiological conditions especially with respect to the effect of efflux transporters which restrict entry of xenobiotics to the brain. Hence, the same authors examined permeability of [3H] naringenin and [14C] quercetin across the blood-brain barrier using an in situ rat brain perfusion model. Test compounds were added to the perfusate injected into rat carotid artery, then their flux into different brain regions was measured. The results demonstrated that both compounds are able to traverse the BBB in vivo, although naringenin permeability was significantly higher. The authors suggested that this difference might be due to quercetin’s affinity to efflux transporters [119]. Similarly, de Boer et al. [120] have demonstrated that long-term feeding of rats with quercetin via diet (~500 mg/kg b.w./day) resulted in the deposition of quercetin and its metabolites isorhamnetin and tamarixetin in brain tissue.

Some flavonoids were demonstrated to cross the blood-brain barrier in a classical in vivo experiment. Following intravenous administration of naringenin to rats (20 mg/kg), the content of its total form (free and glucuronide conjugate) measured after 10 min in brain homogenate was 2.11 µg/g [121]. Unbound hesperetin (0.015 µg/mL) was determined in microdialysates from the brain striata of rats 30 min after intravenous injection of the compound (50 mg/kg) [122]. However, in both reports cited the route of administration was not representative of normal dietary ingestion, hence, in this experimental model processes of absorption, distribution, and gastrointestinal metabolism were not taken into consideration. More reliable are findings of experiments involving oral ingestion of compounds under investigation.

Following oral administration of tangeretin, a citrus flavonoid, (10 mg/kg/day) to rats for 28 days, significant amounts of the compound were found in various regions of the brain: 3.88 ng/mg in the hypothalamus, 2.36 ng/mg in the striatum, and 2.00 ng/mg in the hippocampus [27].

The presence of [3H](−) epigallocatechin gallate (EGCG) was found in the brain of mice administered the compound orally. Duplicate dosing of [3H] EGCG resulted in a 4-fold increase of radioactivity in the brain [123]. Another component of tea, epicatechin, was also shown to have access to the brain. After oral administration to rats of 100 mg/kg/day for 1, 5, and 10 days, epicatechin glucuronide and 3′-*O*-methyl epicatechin glucuronide were detected in the brain, however, their levels were too low for quantification [124]. The same epicatechin metabolite accumulated in the brain of rats after oral administration of grape-derived polyphenolic preparation as well as its components including proanthocyanidins, catechins, and epicatechins [125].

Anthocyanidins, a part of flavonoid family, widely distributed among pigmented fruit and vegetable were demonstrated to cross the blood-brain barrier. Two laboratories reported that anthocyanidins could enter the brain following dietary intake. In the experiment carried out by Talavera et al. [126] rats received a diet supplemented with 15 g blackberry extract per kg diet (14.8 mmol anthocyanidins per kg) for 15 days. The predominant compound found in the brain was cyanidin-3-glucoside which content was higher in the brain than in plasma (0.21 mmol/g tissue vs. 0.15 mmol/mL) [126]. Following a 10-week dietary supplementation with 2% blueberry extract, several anthocyanins were found in the brain of rats in unmetabolized forms. The main compounds identified in the hippocampus and the cortex were a galactoside form of cyanidin and malvidin as well as malvidin-3-*O*-β-arabinose [127].

Kalt et al. [128] reported the findings of the experiment in which pigs were fed diets supplemented with 1, 2, and 4% w/w of freeze-dried blueberries for four weeks. Eleven intact anthocyanidins were detected in the cortex and cerebellum, their total concentration was 0.88 and 0.66 pm/g tissue, respectively. The authors suggest that the consumption of low doses of flavonoids can lead to their accumulation in some tissues including brain even when they are not detected in the circulation [128].

Furthermore, other investigators have shown that ten-day treatment with a combination of polyphenol preparations from grapes or resveratrol led to the accumulation of proanthocyanidins and quercetin glucuronides (Gluc), including catechin-*O*-β-Gluc, 3′-*O*-methyl-catechin-*O*-β-Gluc, epicatechin-*O*-β-Gluc, 3′-*O*-methyl-epicatechin-*O*-β-Gluc, quercetin-3-*O*Gluc, *O*-methyl-quercetin-Gluc, as well as free glycosides such as malvidin-3-*O*-glucoside (Glc), petunidin-Glc, delphinidin-Glc, peonidin-Glc, cyanidin-Glc, malvidin-3-*O*-Glc, petunidin-3-*O*-Glc, delphinidin-3-*O*-Glc, peonidin-3-*O*-Glc, and cyanidin-3-*O*-Glc in the brain of rats. Resveratrol was distributed into the brain, both in unchanged form and conjugated with glucuronic acid [129]. Chen et al. [130] reported that in pigs treated with polyphenols from apple/grape seed and bilberry extracts, metabolites such as catechin/epicatechin-glucuronides, 3′Me*O*-catechin/epicatechin-glucuronides, and quercetin glucuronides were accumulated in the cerebellum, brain stem, medial frontal cortex, hippocampus, hypothalamus, and amygdala, whereas glycosides of anthocyanidins, such as petunidin, malvidin, cyanidin, and peonidin were found only in the first three brain regions.

Besides the above-mentioned feeding with enriched foods, absorption and distribution of purified anthocyanidins was also evidenced. Passamonti et al. [131] introduced surgically a single dose of anthocyanidins extracted from grapes (8 mg/kg b.w.) into the stomach of rats. After 10 min, intact anthocyanidins, mainly malvidin-3-glucoside and its p-cumarate ester, were detected in the brain in quantities similar to that in the plasma, namely 192 ng/g and 176 ng/g, respectively [131]. Metabolism and tissue distribution of pelargonidin following oral administration to rats of a single dose of 50 mg/kg b.w. were investigated. Detectable levels of aglycone, 0.16 nmol/g, were found in the brain [132].

## 5. Polyphenols and the Microbiota–Gut–Brain Axis

Recent research has shown that the gut microbiota plays a key role in biotransformation and bioavailability and thus in the biological activities of phytoconstituents, particularly high-molecular-weight polyphenols. In addition, the interaction has been reported to be reciprocal, since not absorbed in the small intestine, oligomeric and polymeric polyphenols can modulate the composition of the colonic gut microbiota by inhibiting some bacterial populations or stimulating others [133]. Some polyphenols, such as flavan-3-ols, proanthocyanidins, and hydrolyzable tannins (ellagitannins), have been demonstrated to exert both selective prebiotic effects and selective antimicrobial effects against pathogenic gut bacteria [134,135]. Furthermore, commensals residing in the gut may exert a beneficial influence on the brain, since they biotransform polyphenols into metabolites with greater neuroprotective activity than their precursor structures. To achieve this health benefit, polyphenols require conversion into low-molecular-weight metabolites able to cross the blood-brain-barrier. In this respect, catabolic reactions involving C-ring opening and C–C bond breaking in polyphenols, carried out by gut microbiota, are crucial for the polyphenol’s activation. This process has been substantially studied in the catabolism of ellagitannins, mainly pomegranate’s punicalagins, to produce different urolithins by *Gordonibacter* spp. [136]. Ellagitannins are hydrolyzed in the upper gastrointestinal tract, releasing ellagic acid, which together with residual parent compounds is metabolized by gut microbiota to urolithins, including urolithins A, B, C, M6, and isourolithin A in the intestine. However, the ability to produce urolithins differs considerably among individuals depending on their colon microbial composition and three (A, B, and 0) human urolithin-producing metabotypes have been described. Metabotype B individuals produce urolithin B and/or isourolithin A and urolithin A (UA), metabotype A generates only urolithin A while those with metabotype 0 do not produce urolithins at all [137]. The absorbed urolithins undergo subsequent phase I and II metabolism including methylation, glucuronidation, and sulfation [138]. The colonic metabolites, due to their anti-inflammatory, antioxidant, and antiglycative activity, have recently attracted considerable interest in the context of neuroprotection [138,139,140]. However, the neuroprotective potential of urolithins have been mainly demonstrated in vitro. Only Yuan et al. [138] studied the neuroprotective effects of urolithins against Alzheimer’s disease in vivo and have shown that methyl-urolithin B protected *Caenorhabditis elegants* against Aβ1–42-induced neurotoxicity and paralysis. Neuroprotective activity of urolithins is also supported by human data. In middle-aged and older adults with mild memory complaints, drinking 8 ounces of punicalagin-rich pomegranate juice for 4 weeks caused a significant improvement in verbal and visual memory that correlated well with plasma urolithin A-glucuronide concentration [141]. Notably, UA has been recently reported to promote mitochondrial health and induce mitophagy in vivo, which has substantial implications for neuroprotection in Parkinson’s disease [142,143]. However, data about pharmacokinetics and tissue distribution, especially into the brain, of urolithins and their conjugates are somewhat scarce. There are only a few available reports in the literature confirming the distribution of urolithin A to the brain. Gasperotti et al. [144] have detected UA in the brains of rats injected with a mixture of polyphenol microbial metabolites including urolithin A. Seeram et al. [145] have found methylated-UA in the brains of mice administered intraperitoneally with UA. Therefore, Da Silva et al. [146] have recommended conducting animal studies using relevant doses of purified urolithins in order to elucidate their pharmacokinetics, pharmacodynamics, and mechanism of neuroprotective action.

## 6. Summary and Conclusions

A lot of in vivo data presented in the current review suggests that many natural polyphenols may protect against the neurodegenerative characteristics of Parkinson disease.

A wide array of assays reflecting the multifactorial etiology of PD has been employed to evidence the neuroprotective activity of the compounds under investigation. As dopamine depletion in the nigrostriatal region is a major pathological aspect of PD, in the majority of studies, the increase in dopamine and its metabolites’ concentrations as well as in the activity, protein, and mRNA levels of tyrosine hydroxylase, the enzyme responsible for DA synthesis, have been demonstrated.

Protection of dopaminergic neurons and attenuation of the loss of TH-positive cells by polyphenols have also been evidenced on the basis of histopathologic and immunohistochemical examination.

The beneficial effects of the compounds tested were supported by the results of behavioral examinations which indicated the improvement of locomotor activity, reduction of apomorphine-induced rotation, reduction in time of turning, improvement of motor balance and coordination, as well as attenuation of bradykinesia.

Multiple mechanisms of the anti-parkinsonian effects of polyphenols have been suggested in the reports cited in the current review. It is well established that oxidative stress and inflammation are significant factors underlying the process of neurodegeneration. Hence, the parameters of oxidative stress, including oxidative damage to essential cellular macromolecules, the content of GSH, as well as the activity of antioxidant enzymes, have been examined, demonstrating the potential of polyphenols to counteract oxidative damage in the striatum. There is abundant evidence that α-synuclein oligomers trigger activation of astrocytes and microglia cells which secrete proinflammatory cytokines leading to neuroinflammation and mitochondrial dysfunction [147]. A number of polyphenols have been shown to inhibit microglial activation accompanied with the suppressed production of TNF-α, IL-6, IL-1β, as well as COX-2, iNOS, and NO. Simultaneously, some of the compounds discussed enhanced the expression of neurotrophic factors such as glial cell-derived neurotrophic factor (GDNF) which is involved in the survival of DA neurons.

Recently, growing attention is being directed towards some molecular pathways underlying the neuroprotective activity of natural compounds in vivo. Many polyphenols have been shown to restore the reduced activities of protein kinases C (PKCs) which play a fundamental role in programmed cell death and cell survival, thus inhibiting apoptotic signaling pathways. These compounds may modulate events associated with apoptosis by lowering the expression of several proapoptotic genes, such as Bax and Bad, and by increasing the level of antiapoptotic Bcl-2 as well as the inhibition of caspases.

Another signaling pathway involved in apoptotic events which was found to be modulated by polyphenols are Jun N-terminal kinases (JNK). JNKs, belonging to the superfamily of MAP kinases, are responsive to stress stimuli such as cytokines or ROS and play a significant role in stimulating apoptosis. As mentioned above, some polyphenols have been shown to reduce activation of JNKs and transcription factor c-Jun which result in the suppression of dopaminergic neurons’ apoptosis. Thus, the JNK pathway might be considered one of the novel therapeutic targets for neurodegenerative diseases [148].

The promising results of the above presented studies make the continuing search for phytochemicals exerting neuroprotective effects on DA neurons and thus retarding their degeneration highly desirable. Especially significant in future investigations will be the understanding of the molecular and cellular pathways involved in the neurodegenerative process, which will enable the identification of potential targets for effective treatments. The ultimate deliverable is also expected from ongoing research on metabolic derivatives of polyphenols, in particular, bioavailable gut microbiota metabolites, which offer a novel therapeutic approach to the treatment of Parkinson’s disease.

## Figures and Tables

**Table 1:** Summary of the neuroprotective effects of polyphenols in PD models.

Compound, Route and Dosage	Species (Sex)	Neurotoxin	Neuroprotective Effects	References
Naringenin (50 mg/kg; p.o.) 4-day pre-treatment	Sprague- Dawley rats (male)	6-OHDA	↑TH-positive cells ↑DA, DOPAC, and HVA	Zbarsky et al. 2005 [14]
Naringenin (70 mg/kg; p.o.) 4-day pre-treatment	C57BL/6 mice (male)	6-OHDA	↑rotational behavior ↑TH-positive neurons ↑DA, DOPAC, and HVA ↑protein expression of NRf2, HO-1, GCLC, GCLM, HO-1 ↑GSH ↓ROS ↑protein expression of JNK and p38	Lou et al. 2014 [15]
Naringin (80 mg/kg; i.p.) 1-day pre-treatment and 6-day post-treatment	C57BL/6 mice (male)	6-OHDA	↑TH-positive neurons ↓Iba1-positive microglia ↓IL-1β positive microglia ↓6-OHDA—inducedp-4E-BP1	Kim et al. 2016 [16]
Hesperidin (50 mg/kg; p.o.) 28-day post-treatment	aged C57BL/6 mice (female)	6-OHDA	↓time spent immobile (antidepressant-like activity) ↑spatial learning and memory skill ↑DA, DOPAC and HVA ↑GSH level and TRAP ↑CAT and GPx activity ↓ROS levels ↓6-OHDA—induced GR activity	Antunes et al. 2014 [17]
Hesperetin (50 mg/kg; p.o.) 1-week post-treatment	Wistar rats (male)	6-OHDA	↑rotational behavior ↑motor balance and coordination ↓MDA ↑GSH ↑CAT ↑Bcl-2 ↓GFAP expression and DNA fragmentation	Kiasalari et al. 2016 [18]
Quercetin (50 mg/kg; i.g.) 4-day pre-treatment	Sprague-Dawley rats (male)	6-OHDA	No effects	Zbarsky et al. 2005 [14]
Quercetin (50–200 mg/kg; i.p.) once a day for one week before and for one week after the 6-OHDA-infusion (100 mg/kg; i.p.) twice a day for one week before and for one week after the 6-OHDA-infusion	Wistar rats (male)	6-OHDA	No effects	Kääriäinen et al. 2008 [19]
Quercetin (30 mg/kg; i.p.) 14-day post-treatment	Sprague-Dawley rats (male)	6-OHDA	↑DA ↑neuronal density of striatum ↑Nissl-stained neurons ↓PCs ↓LPO ↑GSH	Haleagrahara et al. 2011 [20]
Rutin (25 mg/kg; p.o.) 3-week pre-treatment	Wistar rats (male)	6-OHDA	↑motor co-ordination ↑working performance ↑DA and DOPAC ↓TNF-α and IL-1β ↓NO ↓TBARS, H_2_O_2_, and PCs ↑GSH ↑GPx, GR, SOD and CAT activity	Khan et al. 2012 [21]
Troxerutin (150 mg/kg; p.o.) 1-week pre-treatment	Wistar rats (male)	6-OHDA	↑motor function ↑numbers of TH-positive ↑Nissl positive neurons ↑DA and DOPAC ↓MDA, ROS, NO_2_ ↓GFAP ↓TBARS, H_2_O_2_, and PCs ↓DNA fragmentation ↑GPx, GR, SOD and CAT activity	Baluchnejadmojarad et al. 2017 [22]
Myricitrin (60 mg/kg; i.p.) 1-day pre-treatment and 6-day post-treatment	C57BL/6 mice (male)	6-OHDA	↑motor function ↑TH-positive neurons ↓Iba1-positive microglia ↓TNF-α-positive microglia ↑TH activity ↑p-4E-BP1 and p-TH levels ↑mTORC1	Kim et al. 2016 [16]
Myricetin (5 µl of solution 0.5 mg/mL injected into lateral cerebral ventricle) 7-day post-treatment	Wistar rats (female)	6-OHDA	↑TH-positive neurons ↓iron-staining cells ↑DA, DOPAC and HVA ↑TH and GAPDH mRNA	Ma et al. 2007 [23]
Genistein (10 mg/kg; i.p.) a single dose 1 h before surgery	Sprague–Dawley rats (male)	6-OHDA	↑rotational behavior ↑Nissl-stained neurons	Baluchnejadmojarad et al. 2009 [24]
Puerarin (0.12 mg/kg; i.p.) 10-day co-treatment t	Sprague–Dawley rats (male)	6-OHDA	↑TH-positive cells ↑DA, DOPAC and HVA ↓degeneration of DAargic neurons ↓apoptosis (TUNEL assay) ↓Bax protein expression ↑GDNF protein expression	Zhu et al. 2010 [25]
Baicalein (200 mg/kg; i.g.) 1-week pre-treatment	Sprague–Dawley rats (male)	6-OHDA	↓muscle tremor ↑GABA level ↓ GLu level ↓COI mRNA expression ↓GABA-T protein expression ↑GS protein expression	Yu et al. 2012 [26]
Tangeretin (20 mg/kg; p.o.) 4-day pre-treatment	Sprague-Dawley rats (male)	6-OHDA	↑TH-positive cells ↑DA	Datla et al. 2001 [27]
EGCG (1 and 2 mg/kg; p.o.) 14-day pre-treatment	Sprague–Dawley rats (female)	6-OHDA	↓postural abnormalities ↑ability to cross a narrow beam	Leaver et al. 2009 [28]
Pelargonidin (10 and/or 20 mg/kg; p.o.) 1 day before and on the day of surgery.	Wistar rats (male)	6-OHDA	↑rotational behavior ↑SN neurons ↓TBARS	Roghani et al. 2010 [29]
RES (10, 20 and 40 mg/kg; i.g.) 10-week post-treatment	Sprague–Dawley rats (male)	6-OHDA	↑rotational behavior ↓COX-2 and TNF-α mRNA ↓COX-2 protein	Jin et al. 2008 [30]
RES (20 mg/kg; i.g.) 14-day post-treatment treatment	Wistar rats (male)	6-OHDA	↑rotational behavior ↑total nigral cells and DA neurons ↑total antioxidant capacity ↓number of apoptotic nigral cells ↓ROS	Wang et al. 2011 [31]
RES (20 mg/kg; i.p.) 15-day pre-treatment	Wistar rats (male)	6-OHDA	↑rotational behaviour, motor coordination ↑TH-positive cells ↑DA, DOPAC ↓TBARS, PCs ↓PLA2, COX-2 protein ↑activity of GSH, SOD, CAT, GPx, GR ↑Na^+^/K^+^-ATPase activity	Khan et al. 2010 [32]
Piceid (50 mg/kg; p.o.), 14-day co-treatment	Sprague-Dawley rats (male)	6-OHDA	↓motor defects ↓MDA ↑SOD activity	Chen et al. 2015 [33]
CUR (200 mg/kg; i.g.) twice a day, 24-day pre-treatment	Wistar rats (female)	6-OHDA	↑TH-positive cells ↑DA, DOPAC and HVA ↓iron-stained cells	Du et al. 2012 [34]
CUR (200 mg/kg; i.p.) 7-day post-treatment	ICR mice (male)	6-OHDA	↑TH-positive cells ↓GFAP, Iba1 protein ↑SOD1 level	Tripanichkul et al. 2013 [35]
CA (20 mg/kg; p.o.) 3 times per week, 3-week pre-treatment	Wistar rats (male)	6-OHDA	↑locomotor time and distance traveled ↓TBARS ↑GSH ↑GCLC, GCLM, GR, and SOD protein ↓phosphorylated JNK and p38 protein (↓activation) ↑Bcl-2/Bax ↑cleaved caspase-3/caspase-3 and cleaved PARP/PARP	Wu et al. 2015 [36]
SA (20 mg/kg; p.o.) 2-day pre-treatment	Wistar rats (male)	6-OHDA	↑rotational behavior ↑Nissl-stained, TH-positive and total SN neurons ↓iron-stained cells ↓MDA and nitrite level	Zare et al. 2015 [37]
Ellagic acid (50 mg/kg; i.g) 10-day post-treatment	Wistar rats (male)	6-OHDA	↓motor deficiencies ↓IL-1β and TNF-α protein	Farbood et al. 2015 [38]
Delta 9-tetrahydrocannabinol (3 mg/kg; i.p.) 2-week post-treatment	Sprague-Dawley rats (male)	6-OHDA	↑DA ↑TH mRNA and activity	Lastres-Becker et al. 2005 [39]
EGCG (2 and 10 mg/kg; p.o.) 10-day pre-treatment	C57-BL mice (male)	MPTP	↑DA ↑TH level and activity ↓MPTP-induced activity of SOD and CAT	Levites et al. 2001 [40]
EGCG (2 mg/kg; p.o.) 10-day pre-treatment	C57-BL mice (male)	MPTP	↑DA ↑TH level and activity ↓α-synuclein ↓Bcl-2, Bax protein ↑PKCα protein	Mandel et al. 2004 [41]
EGCG (25 mg/kg; p.o.) 1-day pre-treatment and 5-day co-treatment	C57B6 mice (male)	MPTP	↑TH-positive neurons ↑DA, DOPAC and HVA ↑TH activity ↓nNOS mRNAs, nNOS level and activity	Choi et al. 2002 [42]
EGCG (25 mg/kg; p.o.) 7-day post-treatment	C57 black mice (male)	MPTP	↑rotational behaviour ↑DA and DOPAC ↓PCs ↑ferroportin protein	Xu et al. 2017 [43]
EGCG (10 mg/kg; i.g.) 14-day post-treatment	C57/BL6 mice (male)	MPTP	↑DA, DOPAC and HVA ↑Ras expression	Reznichenko et al. 2010 [44]
EGCG (10 , 50 mg/kg; i.p.) 10-day pre-treatment and 4-day co-treatment	C57Bl/6 mice (male)	MPTP	↑TH-positive neurons ↓iNOS expression	Kim et al. 2010 [45]
EGCG (25 , 50 mg/kg/day; i.g.) 1-day pre-treatment and 20-day post-treatment	C57BL/6 mice (male)	MPTP	↓motor coordination ↑TH-positive neurons ↓CD3^+^CD4^+^/CD3^+^CD8^+^ T cells ↓TNF-α and IL-6 protein in plasma	Zhou et al. 2018 [46]
Acacetin (10 mg/kg; p.o) 3-day co-treatment	C57BL/6 mice (male)	MPTP	↓movement impairment ↑TH-positive neurons ↓damage in DAergic cells ↑DA ↓iNOS mRNA and COX-2 mRNA	Kim et al. 2012 [47]
Baicalein (10 mg/kg; i.g.) 5-day co-treatment	C57BL/6 mice (male)	MPTP	↓motor dysfunction ↓MPTP-induced glutamatergic transmission, presynaptic glutamate release and upregulation of synaptic GluR1 subunit	Xue et al. 2014 [48]
Baicalein (1 and 10 mg/kg; i.g.) 7-day pre-treatment	C57BL/6 mice (male)	MPTP	↓motor dysfunction ↑TH-positive neurons ↓GFAP, Iba1 protein ↓phosphorylated ERK and JNK- ↓activation	Lee et al. 2014 [49]
7,8-dihydroxyflavone (5, 20, 40, and 100 mg/kg; i.p) 7-day pre-and 7-day co-treatment	B57/BL mice (male)	MPTP	↑TH-positive neurons ↑TrkB activity ↓caspase-3 protein	Jang et al. 2010 [50]
Tangeretin (10 mg/kg; i.p.) 4-day pre-treatment	C57BL/6 mice (male)	MPTP	↑TH-positive neurons ↑DA ↑GRP-78 protein	Takano et al. 2007 [51]
Nobiletin (10 mg/kg; i.p.) 1-day pre-treatment and 6-day-post-treatment	Sprague Dawley (SD) rats (female)	MPTP	↑TH-positive neurons ↑DA ↓Iba1 protein and IL-1β ↑GDNF protein	Jeong et al. 2015 [52]
Nobiletin (50 mg/kg; i.p.) 14-day post-treatment	C57BL/6 mice (male)	MPTP	↓motor and cognitive impairment ↑Ca^2+^/calmodulin-dependent protein kinase II (CaMKII) ↑DARPP-32, dopamine- and cAMP-regulated phosphoprotein-32	Yabuki et al. 2014 [53]
Naringin (8 and 800 mg/kg; i.p) 1-day pre-and 6-day post-treatment	Sprague Dawley (SD) rats (female)	MPTP	↑TH-positive neurons ↑GDNF protein ↑mTORC1 activity ↓TNF-α protein	Leem et al. 2014 [54]
Kaempferol (25, 50 and 100 mg/kg; p.o) 14-day pre-treatment	C57BL/6 mice (male)	MPTP	↑Motor behavioral ↑TH-positive neurons ↑DA and DOPAC ↓MDA ↑activity of SOD and GPx	Li and Pu 2011 [55]
Quercetin (100, 200 mg/kg; p.o.) 10-day pre-treatment and 4-day co-treatment	C57BL/6 mice (male)	MPTP	↑motor balance and coordination ↑DA ↑GPx, SOD activity ↑Na^+^, K^+^-ATPase ↓4-HNE ↑AChE activity	Lv et al. 2012 [56]
Morin (5, 20, 40, and 100 mg/kg; i.p) 5-day pre-and 5-day co-treatment	B57/BL mice (male)	MPTP	↓cataleptic time (bar test) and ↑ number of steps (drag test) ↑DA ↑TH-positive neurons	Zhang et al. 2010 [57]
Silibinin (1 or 10 mg/kg; i.p.) 5-day co-treatment	C57BL/6 mice (male)	MPTP	↓Motor dysfunction ↓DAergic neuron damage	Lee et al. 2015 [58]
Silibinin (50, 100 mg/kg; i.p.) 5-day co-treatment	Sprague Dawley (female)	MPTP	↑TH-positive neurons ↓IL-1β, TNF-α and iNOS protein	Jung et al. 2014 [59]
Silibinin (100, 200 mg/kg; i.g.) 7-day co-treatment	albino rats of Charles– Foster strain (male)	MPTP	↑spatial memory and locomotor activity ↓mitochondrial complex-I and IV activity ↑mitochondrial complex-II and V activity ↑mitochondrial membrane potential ↓NO, MDA ↑SOD activity ↓caspase-3 and NFκB protein	Geed et al. 2014 [60]
Genistein (10 mg/kg; i.p.) 3-day pre-treatment and 5-day-co-treatment	C57BL/6 mice (male)	MPTP	↑TH-positive neurons ↑DA, DOPAC and HVA ↑TH and DAT mRNA ↓Bcl-2 mRNA	Liu et al. 2008 [61]
Gastrodin (10, 30 and 60 mg/kg; p.o.) 15-day co-treatment	C57BL/6 mice (male)	MPTP	↓bradykinesia and motor impairment ↑TH protein ↓GFAP protein ↑Bcl-2 protein ↓Bax and caspase-3 protein ↓PARP cleavage	Kumar et al. 2013 [62]
Tanshinone I (5 and 10 mg/kg; i.g.) 7-day pre- and co-treatment	C57BL/6 mice (male)	MPTP	↓Motor dysfunction ↑DA, DOPAC and HVA ↑TH-positive neurons ↓Iba1-positive activated microglia cells ↓TNF-α and IL-10 protein	Wang et al. 2015 [63]
Ferulic acid (40 mg/kg; i.g.) 3-day pre-treatment and 4-day co-treatment	C57BL/6 mice (male)	MPTP	↑motor balance and coordination ↓anxiety ↓degeneration of DAargic neurons ↓Bax/Bcl-2 ratio	Nagarajan et al. 2015 [64]
Madecassoside (15, 30 and 60 mg/kg; i.g) 7-day pre-treatment and 14-day co-treatment	Wistar rats m (male)	MPTP	↑limb coordination and limb placing ↑DA, DOPAC and HVA ↓MDA ↑GSH ↑Bcl-2/Bax ratio ↑BDNF protein	Xu et al. 2013 [65]
Curcumin (50 mg/kg; i.p.) 3-times at time points 1, 3, and 7 h post first MPTP injection	ICR mice (male)	MPTP	↑DA ↑density of DA neurons	Vajragupta et al. 2003 [66]
Curcumin (80 mg/kg; i.p.) 7-day co-treatment	Swiss albino mice (male)	MPTP	↑DA and DOPAC ↓MAO B activity	Rajeswari et al. 2008 [67]
Curcumin (150 mg/kg; p.o.) 1-week pre-treatment	C57BL/6 mice (male)	MPTP	↑motor performance (rotarod test) ↓steep reduction intotal ambulation time (open field test) ↑DA, DOPAC and HVA ↓GFAP over-expression ↓IL-6 and TNF-α protein ↓iNOS protein and NO content	Ojha et al. 2012 [68]
Ginsenoside Rg1 (5, 10 and 20 mg/kg; i.p.) 3-day pre-treatment and 5-day co-treatment	C57-BL mice (male)	MPTP	↑numbers of TH-positive and Nissl positive neurons in SN ↓TUNEL positive neurons in SN ↑GSH ↓MPTP-induced SOD activity ↓MPTP-induced phosphor—JNK and phospho-c-Jun level	Chen et al. 2005 [69]
Piceid (100 and 200 mg/kg; p.o.) 7-day co-treatment and 7-day post-treatment	C57BL/6 mice (male)	MPTP	↑locomotor activity ↑TH-positive neurons ↑SOD activity ↓MDA level ↑p-Akt expression ↓activated caspase 3 expression	Chen et al. 2015 [33]
TF (10 mg/kg; i.g.) 35-day co-treatment	C57BL/6 mice (male)	MPTP/p	↑Locomotor activity ↑TH- and DAT-positive neurons ↓caspase-3, 8, and 9 protein	Anandhan et al. 2012 [70]
TF (10 mg/kg; i.g.) 3-day pre-treatment and 4-day co-treatment	C57BL/6 mice (male)	MPTP	↑Locomotor activity ↑DAT-positive neurons ↓TBARS, ↑ GSH ↑SOD, CAT and GPx activity ↓MAO-B activity	Anandhan et al. 2012b [71]
TF (10 mg/kg; i.g.) 35-day co-treatment	C57BL/6 mice (male)	MPTP/p	↓Akinesia, catalepsy ↓IL-1β, TNF-α, IL-6 as well as MPTP-induced IL-4 and IL-10 ↓GFAP and COX-2 protein ↑Bcl-2 protein ↓Bax protein	Anandhan et al. 2013 [72]
Apigenin (5, 10 and 20 mg/kg; p.o.), luteolin (10 and 20 mg/kg; p.o.) 5-day pre-treatment and 21-day co-treatment	Swiss-albino mice (male)	MPTP/p	↑Locomotor activity ↑TH-positive neurons ↓MDA, ↑GSH ↑SOD and CAT activity ↓GFAP and TNF-α protein ↑BDNF protein ↓MAO-B activity	Patil et al. 2014 [73]
SA (20 mg/kg; i.g.) 35-day co-treatment	C57BL/6 mice (male)	MPTP/p	↓impairment of motor coordination ↑TH- and DAT-positive neurons ↑TH, DAT and VMAT2 protein ↑DA, DOPAC and HVA ↓TBARS, ↑ GSH ↑SOD, CAT and GPx activity ↓IL-1β, TNF-α protein ↓COX-2 protein	Rekha et al. 2014 [74]
Quercetin (25, 50, 75 mg/kg; i.p.) 4-day post-treatment	Sprague Dawley rats (male)	ROT	↑rotational behavior ↑TH-positive neurons ↓TUNEL positive neurons ↑DA ↑complex-I activity ↑GSH, GSSG ↓ROT-induced activity of SOD and CAT	Karuppagounder et al. 2013 [75]
CUR (20 mg/kg; i.g.) 50-day co-treatment	Lewis rats (male)	ROT	↓postural impairment ↑motor coordination ↑TH activity ↓MDA, ↑ GSH ↑HO-1 and NQO1 protein ↑Akt/Nrf2 phosphorylation (activation)	Cui et al. 2016 [76]
Piceid (80 mg/kg; i.g.) 5-week co-treatment	Sprague Dawley rats (male)	ROT	↓postural impairment ↑motor coordination ↑TH-positive neurons ↑ATP ↑Trx, GSH ↑SOD activity	Chen et al. 2015 [33]
FA (80 mg/kg; i.g.) 4-week co-treatment	Wistar rats (male)	ROT	↑TH-positive neurons ↑SOD and CAT activity ↓MDA, ↑ GSH ↓GFAP and Iba1 protein ↓IL-1β, TNF-α, IL-6 protein ↓COX-2 and iNOS protein	Ojha et al. 2015 [77]
Sesamol (15 mg/kg; p.o.) naringenin(10 mg/kg; p.o.) 10-day post-treatment	Wistar rats (male)	ROT	↑motor coordination ↑body weight ↓degenerated neurons ↑TH and ubiquitin expression ↑parkin, CHIP, and DJ1 expression ↓caspase-9 and 3 protein ↑Hsp70 and Hsp90 protein	Sonia Angeline et al. 2013 [78]
EGCG (10 mg/kg; i.p.) 24 h before LPS and continues for 7 days	Sprague Dawley rats (male)	LPS	↑TH-positive neurons ↑DA ↓TNF-α and nitrite	AL-amri et al. 2013 [79]
CUR (50 mg/kg; i.p.) 10-day treatment beginning 5 days prior to Hcy	Lewis rats (male)	Hcy	↑Locomotor Activity ↑Nissl-stained neurons ↑Bcl-2 protein and ↓ Bax protein ↓Bax/Bcl-2	Mansouri et al. 2012 [80]

↑ = increase, ↓ = decrease.

## Abbreviations

4-HNE	4-hydroxynonenal
6-OHDA	6-Hydroxydopamine
AChE	Acetylcholinesterase
AKT	serine/threonine protein kinase
ARE	Antioxidant responsive element
Bax	Bcl-2-like protein 4
Bcl-2	B-cell lymphoma 2
BDNF	Brain-derived neurotrophic factor.
b.w.	Body weight
CaMKII	Ca^2+^/calmodulin-dependent protein kinase II
Casp-3	Caspase-3
Casp-8	Caspase-8
Casp-9	Caspase-9
CAT	Catalase
CHIP	C terminus Hsp70 interacting protein
CNS	Central nervous system
COI	Cytochrome c oxidase I
COX-2	Cyclooxygenase-2
CUR	Curcumin
DA	Dopamine
DARPP-32	Dopamine- and cAMP-regulated phosphoprotein
DAT	Dopamine transporter
DJ-1	Protein deglycase
DOPAC	3,4-dihydroxyphenylacetic acid
EC	(−)-Epicatechin
ECG	Epicatechin gallate
EGC	(−)-Epigallocatechin
EGCG	Epigallocatechin-3-gallate
ERK	Extracellular signal-regulated kinase protein-serine/threonine kinase
FA	Ferulic acid
GAPDH	Glyceraldehyde 3-phosphate dehydrogenase
GABA	Gamma-aminobutyric acid
GCLC	Glutamate cysteine ligase catalytic subunit
GCLM	Glutamate cysteine ligase modifier subunit
GDNF	Glial cell-derived neurotrophic factor
GFAP	Glial fibrillary acidic protein
GLu	Glutamate (or Glutamic acid)
GPx	Glutathione peroxidase
GR	Glutathione reductase
GRP-78	78 kDa Glucose-regulated protein
GS	Glutamine synthetase
GSH	Reduced glutathione
GSSG	Oxidized glutathione
GTPs	Green tea polyphenols
Hcy	Homocysteine
HO-1	Heme oxygenase-1
Hsp70s	The 70 kDa heat shock proteins
HVA	Homovanillic acid
Iba1	Ionised calcium binding adapter molecule 1
i.p.	Intraperitoneally
IL-1β	Interleukin 1 beta
IL-6	Interleukin 6
IL-10	Interleukin 10
iNOS	Inducible nitric oxide synthase
JNK	c-Jun *N*-terminal kinase
JSE	*Juglandis Semen* extract
L-DOPA	l-3,4-dihydroxyphenylalanine
LPS	Lipopolysaccharide
MAO	Monoamine oxidase
MAPK	Mitogen-activated protein kinase
MB	Maneb
MDA	Malondialdehyde
MMPs	N-methyl-4-phenyl-1,2,3,6-tetrahydropyridine
MPTP	Mitochondrial transmembrane potentials
mTORC1	Mammalian target of rapamycin complex 1
NF-кB	Nuclear factor-кB
nNOS	Nuclear nitric oxide synthase
NO	Nitric oxide
Nrf2	Nuclear factor (erythroid-derived 2)-like 2
NQO1	NAD(P)H: quinone oxidoreductase 1
(p)	probenocid
p-4E-BP1	Phosphorylated 4E-binding protein 1
p38	MAP Kinase (MAPK), CSBP Cytokinin-Specific Binding Protein or RK
PARP	Poly(ADP-ribose) polymerase
PCA	Protocatechuic acid
PCs	Protein carbonyls
PD	Parkinson disease
PGE2	Prostaglandin E2
PI3K	phosphoinositide 3-kinase
PKC	Protein kinase C
PLA2	Phospholipases A2
p.o.	Orally
PQ	Resveratrol
RES	Paraquat
ROS	Reactive oxygen species
ROT	Rotenone
SA	Syringic acid
SIL	Silibinin
SNpc	Substantia nigra pars compacta
SOD	Superoxide dismutase
TBARS	Thiobarbituric acid reactive substances
TH	Tyrosine hydroxylase
TNF-α	Tumor necrosis factor alpha
TRAP	Total reactive antioxidant potential
Trx	Thioredoxin
TF	Theaflavin
TPs	Tea polyphenols
TrkB	Tropomyosin receptor kinase B
TUNEL	Terminal deoxynucleotidyl transferase dUTP nick end labeling
UA	Urolithin A
VMAT2	Vesicular monoamine transporter-2
